# (8-Bromo-2,7-dimeth­oxy-1-naphth­yl)(4-chloro­phenyl)methanone

**DOI:** 10.1107/S1600536810009463

**Published:** 2010-03-20

**Authors:** Ryosuke Mitsui, Atsushi Nagasawa, Shoji Watanabe, Akiko Okamoto, Noriyuki Yonezawa

**Affiliations:** a Department of Organic and Polymer Materials Chemistry, Tokyo University of Agriculture & Technology, 2-24-16 Naka-machi, Koganei, Tokyo 184-8588, Japan

## Abstract

In the title compound, C_19_H_14_BrClO_3_, the naphthalene ring system and the benzene ring make a dihedral angle of 77.36 (10)°. The conformation around the central C=O group is such that the C=O bond vector forms a larger angle to the plane of the naphthalene ring system than to the plane of the benzene ring, *viz.* 75.73 (15)° *versus* 2.33 (17)°. In the crystal structure, a π–π inter­action is formed between naphthalene ring systems, with a centroid–centroid distance of 3.8363 (14) Å and a lateral offset of 1.606 Å. Inter­molecular C—H⋯Br and C—H⋯O hydrogen bonds and a C—H⋯π contact are present in the crystal structure.

## Related literature

For the structures of closely related compounds, see: Mitsui *et al.* (2009 ▶, 2010 ▶); Mitsui, Nakaema, Noguchi, Okamoto & Yonezawa (2008 ▶); Mitsui, Nakaema, Noguchi & Yonezawa (2008 ▶).
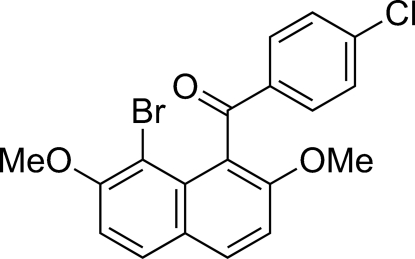


## Experimental

### 

#### Crystal data

C_19_H_14_BrClO_3_
*M* *_r_* = 405.66
Monoclinic, 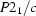

*a* = 15.0867 (3) Å
*b* = 8.72313 (16) Å
*c* = 13.5894 (3) Å
β = 108.536 (1)°
*V* = 1695.64 (6) Å^3^
*Z* = 4
Cu *K*α radiation
μ = 4.88 mm^−1^
*T* = 193 K
0.50 × 0.40 × 0.20 mm

#### Data collection

Rigaku R-AXIS RAPID diffractometer
Absorption correction: numerical (*NUMABS*; Higashi, 1999 ▶) *T*_min_ = 0.160, *T*_max_ = 0.614
26838 measured reflections
3092 independent reflections
2845 reflections with *I* > 2σ(*I*)
*R*_int_ = 0.071

#### Refinement

*R*[*F* ^2^ > 2σ(*F* ^2^)] = 0.034
*wR*(*F* ^2^) = 0.086
*S* = 1.07
3092 reflections
220 parameters
H-atom parameters constrained
Δρ_max_ = 0.45 e Å^−3^
Δρ_min_ = −0.43 e Å^−3^

### 

Data collection: *PROCESS-AUTO* (Rigaku, 1998 ▶); cell refinement: *PROCESS-AUTO*; data reduction: *CrystalStructure* (Rigaku/MSC, 2004 ▶); program(s) used to solve structure: *SIR2004* (Burla *et al.*, 2005 ▶); program(s) used to refine structure: *SHELXL97* (Sheldrick, 2008 ▶); molecular graphics: *ORTEPIII* (Burnett & Johnson, 1996 ▶); software used to prepare material for publication: *SHELXL97*.

## Supplementary Material

Crystal structure: contains datablocks I, global. DOI: 10.1107/S1600536810009463/is2530sup1.cif

Structure factors: contains datablocks I. DOI: 10.1107/S1600536810009463/is2530Isup2.hkl

Additional supplementary materials:  crystallographic information; 3D view; checkCIF report

## Figures and Tables

**Table 1:** Hydrogen-bond geometry (Å, °) *Cg*1 is the centroid of the C1–C5/C10 ring.

*D*—H⋯*A*	*D*—H	H⋯*A*	*D*⋯*A*	*D*—H⋯*A*
C4—H4⋯O1^i^	0.95	2.49	3.366 (3)	154
C19—H19*A*⋯Br1^ii^	0.98	2.92	3.871 (3)	165
C19—H19*C*⋯O1^iii^	0.98	2.53	3.211 (3)	126
C19—H19*B*⋯*Cg*1^iv^	0.98	2.70	3.509 (3)	140

Symmetry codes: (i) 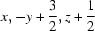; (ii) 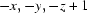; (iii) 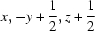; (iv) 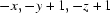.

**Fig. 1.**
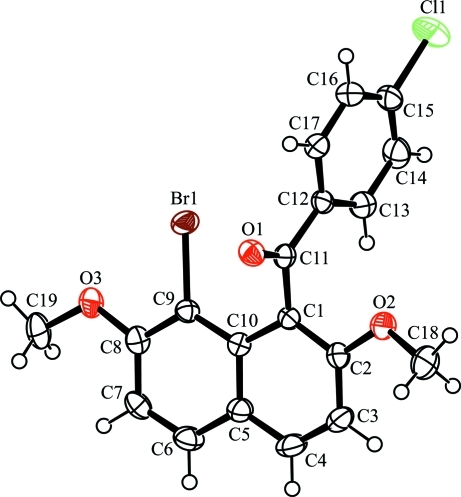
The molecular structure of compound (I), showing 50% probability displacement ellipsoids.

**Fig. 2.**
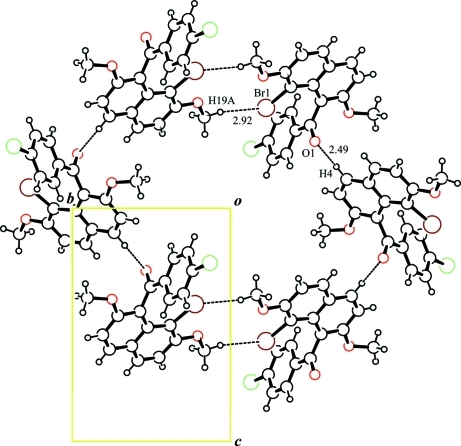
Partial packing diagram of compound (I), viewed down the a axis. Intermolecular C—H···Br and C—H···O hydrogen bonds are shown as dashed lines.

**Fig. 3.**
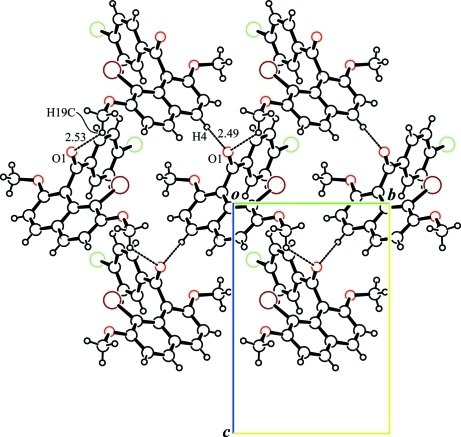
Partial packing diagram of compound (I), viewed down the a axis. Intermolecular C—H···O hydrogen bonds are shown as dashed lines.

**Fig. 4.**
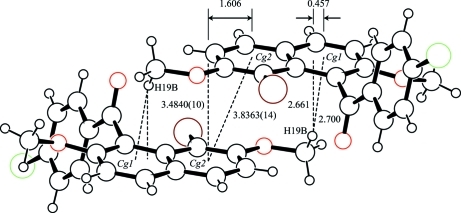
The arrangement of the molecules, viewed in an oblique direction. Intermolecular C—H···π and π–π interactions are shown as dashed lines.

## supplementary crystallographic information

### Comment

Recently, we reported the crystal structures of 1-aroylated 2,7-dimethoxynaphthalenes, 1-(4-chlorobenzoyl)-2,7-dimethoxynaphthalene (Mitsui, Nakaema, Noguchi, Okamoto & Yonezawa, 2008), (4-chlorophenyl)(2-hydroxy-7-methoxynaphthalen-1-yl)methanone (Mitsui, Nakaema, Noguchi & Yonezawa, 2008), (4-chlorophenyl)(2-ethoxy-7-methoxynaphthalen-1-yl)methanone (Mitsui *et al.*, 2009) and 1-bromo-8-(4-chlorobenzoyl)-7-hydroxy-2-methoxynaphthalene (Mitsui *et al.*, 2010). As a part of our ongoing studies on the synthesis and crystal structure analysis of aroylated naphthalene derivatives, we prepared and analysed the structure of crystal of 1-bromo-8-(4-chlorobenzoyl)-2,7-dimethoxynaphthalene, (I). The title compound was prepared by methylation of 1-bromo-8-(4-chlorobenzoyl)-7-hydroxy-2-methoxynaphthalene with dimethyl sulfonate.

An *ORTEPIII* (Burnett & Johnson, 1996) plot of (I) is shown in Fig. 1. In the molecule of (I), the dihedral angle between the benzene ring (C12–C17) and the naphthalene ring (C1–C10) is 77.36 (10)°. The C═O bond vector and the least-squares plane of the benzene ring are almost coplanar [2.33 (17)°]. By contrast, the C═O bond vector and the least-squares plane of the naphthalene ring are largely twisted [75.73 (15)°]. The conformation of these groups resembles to that of 1-(4-chlorobenzoyl)-2,7-dimethoxynaphthalene.

In the crystal structure, all H atoms belonging the methoxy group in the 7-position of naphthalene ring, interact with adjacent molecule constructing intermolecular C—H···Br, C—H···O hydrogen bonds and C—H···π contact, respectively (Table 1). H19A and Br1 interact with each other [H19A···Br1 = 2.92 Å] along the *b* axis (Fig. 2). H19C interacts with the carbonyl oxygen [H19C···O1 = 2.53 Å] along the *b* axis (Fig. 3). The carbonyl oxygen also interacts with naphthalene ring hydrogen [O1···H4 = 2.49 Å] along the *c* axis (Figs. 2 and 3). The methoxy group acts as a hydrogen-bond donor and the π system of the naphthalene ring [C1/C2/C3/C4/C5/C10 ring (with centroid *Cg*1)] of an adjacent molecule acts as an acceptor, *viz.* C19—H19B···π (Fig. 4 and Table 1). Additionally, the π systems of the C5–C10 ring (with centroid *Cg*2) in the naphthalene group are exactly parallel. The perpendicular distance between these aromatic rings is 3.4840 (10) Å. The centroid–centroid distance between the parallel aromatic rings is 3.8363 (14) Å, and the lateral offsets are 1.606 Å, indicating the presence of a π–π interaction (Fig. 4).

### Experimental

1-Bromo-8-(4-chlorobenzoyl)-7-hydroxy-2-methoxynaphthalene (1.56 g, 4.0 mmol) was dissolved in acetone (5.0 ml) and aqueous 0.4 M NaOH (5.0 ml). Then, dimethyl sulfate (0.78 ml, 8.0 mmol) was added and the reaction mixture was stirred for 6 h at room temperature. The mixture was concentrated by evaporation and poured into a mixture of H_2_O (10 ml) and CHCl_3_ (10 ml), and the aqueous layer was extracted with CHCl_3_ (3 × 10 ml). The combined organic layers were washed with brine (3 × 30 ml), and dried over MgSO_4_ overnight. The solvent was removed in vacuo and the crude material was purified by recrystallization from CHCl_3_/hexane to give the title compound as colorless platelets (m.p. 447.0–447.5 K, yield 1.43 g, 88%).

Spectroscopic Data: ^1^H NMR (300 MHz, CDCl_3_) δ 7.88–7.79 (m, 4H), 7.37 (d, 2H), 7.21–7.15 (m, 2H), 3.95 (s, 3H), 3.75 (s, 3H); ^13^C NMR (75 MHz, CDCl_3_) δ 196.5, 157.2, 155.7, 138.7, 138.6, 132.2, 131.9, 130.4, 130.1, 128.7, 126.3, 122.5, 111.8, 111.6, 104.9, 57.0, 56.8; IR (KBr): 1665, 1613, 1506, 1273, 1043, 827; HRMS (*m*/*z*): [M + H]^+^ calcd for C_19_H_15_BrClO_3_, 404.9893 found, 404.9862. Anal. Calcd for C_19_H_14_BrClO_3_: C 56.25, H 3.48. Found: C 56.48, H 3.42.

### Refinement

All the H atoms could be located in a difference Fourier map. The C-bound H atoms were subsequently refined as riding atoms, with C—H = 0.95 (aromatic) and 0.98 (methyl) Å, and with *U*_iso_(H) = 1.2*U*_eq_(C).

### Crystal data

**Table:**

C_19_H_14_BrClO_3_	*F*(000) = 816
*M**_r_* = 405.66	*D*_x_ = 1.589 Mg m^−^^3^
Monoclinic, *P*2_1_/*c*	Melting point = 447.0–447.5 K
Hall symbol: -P 2ybc	Cu *K*α radiation, λ = 1.54187 Å
*a* = 15.0867 (3) Å	Cell parameters from 25305 reflections
*b* = 8.72313 (16) Å	θ = 3.1–68.2°
*c* = 13.5894 (3) Å	µ = 4.88 mm^−^^1^
β = 108.536 (1)°	*T* = 193 K
*V* = 1695.64 (6) Å^3^	Platelet, colorless
*Z* = 4	0.50 × 0.40 × 0.20 mm

### Data collection

**Table:**

Rigaku R-AXIS RAPID diffractometer	3092 independent reflections
Radiation source: rotating anode	2845 reflections with *I* > 2σ(*I*)
graphite	*R*_int_ = 0.071
Detector resolution: 10.00 pixels mm^-1^	θ_max_ = 68.2°, θ_min_ = 3.1°
ω scans	*h* = −18→18
Absorption correction: numerical (*NUMABS*; Higashi, 1999)	*k* = −10→10
*T*_min_ = 0.160, *T*_max_ = 0.614	*l* = −16→15
26838 measured reflections	

### Refinement

**Table:**

Refinement on *F*^2^	Secondary atom site location: difference Fourier map
Least-squares matrix: full	Hydrogen site location: inferred from neighbouring sites
*R*[*F*^2^ > 2σ(*F*^2^)] = 0.034	H-atom parameters constrained
*wR*(*F*^2^) = 0.086	*w* = 1/[σ^2^(*F*_o_^2^) + (0.036*P*)^2^ + 0.9139*P*] where *P* = (*F*_o_^2^ + 2*F*_c_^2^)/3
*S* = 1.07	(Δ/σ)_max_ = 0.001
3092 reflections	Δρ_max_ = 0.45 e Å^−^^3^
220 parameters	Δρ_min_ = −0.43 e Å^−^^3^
0 restraints	Extinction correction: *SHELXL97* (Sheldrick, 2008), Fc^*^=kFc[1+0.001xFc^2^λ^3^/sin(2θ)]^-1/4^
Primary atom site location: structure-invariant direct methods	Extinction coefficient: 0.0034 (2)

### Special details

**Table:**

Geometry. All esds (except the esd in the dihedral angle between two l.s. planes) are estimated using the full covariance matrix. The cell esds are taken into account individually in the estimation of esds in distances, angles and torsion angles; correlations between esds in cell parameters are only used when they are defined by crystal symmetry. An approximate (isotropic) treatment of cell esds is used for estimating esds involving l.s. planes.
Refinement. Refinement of *F*^2^ against ALL reflections. The weighted *R*-factor wR and goodness of fit *S* are based on *F*^2^, conventional *R*-factors *R* are based on *F*, with *F* set to zero for negative *F*^2^. The threshold expression of *F*^2^ > σ(*F*^2^) is used only for calculating *R*-factors(gt) etc. and is not relevant to the choice of reflections for refinement. *R*-factors based on *F*^2^ are statistically about twice as large as those based on *F*, and *R*- factors based on ALL data will be even larger.

### Fractional atomic coordinates and isotropic or equivalent isotropic displacement parameters (Å^2^)

**Table:**

	*x*	*y*	*z*	*U*_iso_*/*U*_eq_	
Br1	0.147755 (18)	0.22446 (3)	0.41929 (2)	0.03662 (13)	
Cl1	0.50868 (6)	0.13053 (10)	0.23975 (8)	0.0709 (3)	
O1	0.14525 (10)	0.53370 (19)	0.27564 (12)	0.0318 (4)	
O2	0.32367 (14)	0.7494 (2)	0.39704 (16)	0.0411 (5)	
O3	0.03824 (12)	0.2074 (2)	0.55691 (15)	0.0372 (4)	
C1	0.23238 (14)	0.5731 (2)	0.44962 (17)	0.0248 (5)	
C2	0.27916 (16)	0.7120 (3)	0.4674 (2)	0.0307 (5)	
C3	0.27780 (19)	0.8080 (3)	0.5498 (2)	0.0401 (6)	
H3	0.3116	0.9017	0.5619	0.048*	
C4	0.2273 (2)	0.7647 (3)	0.6119 (2)	0.0417 (6)	
H4	0.2251	0.8309	0.6666	0.050*	
C5	0.17794 (16)	0.6246 (3)	0.59797 (19)	0.0313 (5)	
C6	0.12237 (18)	0.5854 (3)	0.66060 (19)	0.0375 (6)	
H6	0.1192	0.6544	0.7135	0.045*	
C7	0.07338 (16)	0.4519 (3)	0.64755 (19)	0.0339 (6)	
H7	0.0346	0.4304	0.6889	0.041*	
C8	0.08055 (15)	0.3461 (3)	0.57235 (18)	0.0294 (5)	
C9	0.13469 (15)	0.3817 (3)	0.51018 (17)	0.0253 (5)	
C10	0.18258 (14)	0.5234 (2)	0.51737 (16)	0.0243 (5)	
C11	0.21803 (15)	0.5047 (2)	0.34374 (17)	0.0251 (5)	
C12	0.29276 (15)	0.4137 (2)	0.32081 (18)	0.0264 (5)	
C13	0.37831 (16)	0.3841 (3)	0.39620 (19)	0.0325 (5)	
H13	0.3907	0.4235	0.4644	0.039*	
C14	0.44561 (17)	0.2970 (3)	0.3718 (2)	0.0406 (6)	
H14	0.5043	0.2775	0.4228	0.049*	
C15	0.42620 (18)	0.2396 (3)	0.2732 (2)	0.0402 (7)	
C16	0.34149 (19)	0.2680 (3)	0.1967 (2)	0.0397 (6)	
H16	0.3294	0.2279	0.1287	0.048*	
C17	0.27547 (16)	0.3553 (3)	0.22136 (19)	0.0321 (5)	
H17	0.2174	0.3758	0.1697	0.039*	
C18	0.3499 (2)	0.9051 (3)	0.3903 (3)	0.0476 (7)	
H18A	0.3716	0.9176	0.3300	0.057*	
H18B	0.4004	0.9329	0.4535	0.057*	
H18C	0.2959	0.9718	0.3827	0.057*	
C19	−0.01877 (17)	0.1655 (3)	0.6194 (2)	0.0425 (7)	
H19A	−0.0466	0.0645	0.5978	0.051*	
H19B	−0.0685	0.2416	0.6104	0.051*	
H19C	0.0199	0.1618	0.6925	0.051*	

### Atomic displacement parameters (Å^2^)

**Table:**

	*U*^11^	*U*^22^	*U*^33^	*U*^12^	*U*^13^	*U*^23^
Br1	0.05100 (19)	0.02421 (18)	0.0425 (2)	−0.00965 (10)	0.02598 (14)	−0.00643 (10)
Cl1	0.0531 (4)	0.0667 (5)	0.1010 (7)	0.0177 (4)	0.0360 (4)	−0.0234 (5)
O1	0.0301 (8)	0.0363 (9)	0.0288 (9)	0.0025 (7)	0.0094 (7)	0.0005 (7)
O2	0.0518 (11)	0.0298 (9)	0.0537 (13)	−0.0136 (8)	0.0336 (10)	−0.0043 (8)
O3	0.0405 (9)	0.0338 (10)	0.0443 (11)	−0.0086 (7)	0.0236 (8)	0.0042 (8)
C1	0.0244 (10)	0.0233 (11)	0.0284 (12)	0.0000 (8)	0.0108 (9)	−0.0009 (9)
C2	0.0310 (11)	0.0301 (13)	0.0351 (14)	−0.0043 (9)	0.0163 (10)	−0.0003 (10)
C3	0.0476 (14)	0.0312 (13)	0.0453 (16)	−0.0136 (11)	0.0201 (12)	−0.0121 (12)
C4	0.0554 (16)	0.0370 (14)	0.0369 (15)	−0.0104 (12)	0.0207 (13)	−0.0157 (12)
C5	0.0358 (12)	0.0307 (12)	0.0296 (13)	−0.0025 (10)	0.0136 (10)	−0.0030 (10)
C6	0.0471 (14)	0.0396 (14)	0.0305 (14)	0.0011 (11)	0.0191 (11)	−0.0049 (11)
C7	0.0357 (12)	0.0410 (14)	0.0314 (13)	0.0029 (10)	0.0194 (10)	0.0055 (11)
C8	0.0280 (10)	0.0283 (12)	0.0322 (13)	0.0015 (9)	0.0099 (9)	0.0075 (10)
C9	0.0287 (10)	0.0252 (11)	0.0233 (11)	0.0003 (9)	0.0100 (9)	0.0009 (9)
C10	0.0248 (10)	0.0241 (11)	0.0236 (11)	0.0006 (8)	0.0069 (8)	0.0017 (9)
C11	0.0297 (11)	0.0209 (11)	0.0276 (12)	−0.0044 (8)	0.0131 (9)	0.0029 (9)
C12	0.0293 (11)	0.0219 (11)	0.0324 (13)	−0.0019 (8)	0.0160 (9)	0.0009 (9)
C13	0.0356 (12)	0.0310 (12)	0.0316 (13)	−0.0006 (10)	0.0116 (10)	0.0012 (10)
C14	0.0308 (12)	0.0351 (14)	0.0541 (17)	0.0055 (10)	0.0110 (12)	0.0050 (13)
C15	0.0366 (13)	0.0287 (13)	0.062 (2)	0.0007 (10)	0.0258 (13)	−0.0069 (12)
C16	0.0423 (14)	0.0349 (14)	0.0468 (17)	−0.0038 (11)	0.0212 (13)	−0.0119 (12)
C17	0.0338 (11)	0.0291 (12)	0.0357 (14)	−0.0050 (9)	0.0142 (10)	−0.0047 (10)
C18	0.0543 (16)	0.0321 (14)	0.0622 (19)	−0.0114 (12)	0.0269 (14)	0.0052 (13)
C19	0.0357 (13)	0.0438 (16)	0.0538 (17)	−0.0021 (11)	0.0223 (12)	0.0171 (14)

### Geometric parameters (Å, °)

**Table:**

Br1—C9	1.898 (2)	C7—H7	0.9500
Cl1—C15	1.738 (3)	C8—C9	1.384 (3)
O1—C11	1.217 (3)	C9—C10	1.419 (3)
O2—C2	1.371 (3)	C11—C12	1.490 (3)
O2—C18	1.426 (3)	C12—C17	1.389 (3)
O3—C8	1.353 (3)	C12—C13	1.393 (3)
O3—C19	1.435 (3)	C13—C14	1.390 (4)
C1—C2	1.385 (3)	C13—H13	0.9500
C1—C10	1.428 (3)	C14—C15	1.372 (4)
C1—C11	1.508 (3)	C14—H14	0.9500
C2—C3	1.403 (4)	C15—C16	1.389 (4)
C3—C4	1.359 (4)	C16—C17	1.377 (3)
C3—H3	0.9500	C16—H16	0.9500
C4—C5	1.412 (3)	C17—H17	0.9500
C4—H4	0.9500	C18—H18A	0.9800
C5—C6	1.414 (3)	C18—H18B	0.9800
C5—C10	1.426 (3)	C18—H18C	0.9800
C6—C7	1.360 (4)	C19—H19A	0.9800
C6—H6	0.9500	C19—H19B	0.9800
C7—C8	1.406 (4)	C19—H19C	0.9800
			
C2—O2—C18	118.6 (2)	O1—C11—C1	117.7 (2)
C8—O3—C19	118.6 (2)	C12—C11—C1	121.43 (18)
C2—C1—C10	119.8 (2)	C17—C12—C13	119.4 (2)
C2—C1—C11	115.1 (2)	C17—C12—C11	118.6 (2)
C10—C1—C11	123.34 (18)	C13—C12—C11	122.0 (2)
O2—C2—C1	114.9 (2)	C14—C13—C12	120.2 (2)
O2—C2—C3	123.3 (2)	C14—C13—H13	119.9
C1—C2—C3	121.7 (2)	C12—C13—H13	119.9
C4—C3—C2	118.9 (2)	C15—C14—C13	119.1 (2)
C4—C3—H3	120.5	C15—C14—H14	120.4
C2—C3—H3	120.5	C13—C14—H14	120.4
C3—C4—C5	122.2 (3)	C14—C15—C16	121.7 (2)
C3—C4—H4	118.9	C14—C15—Cl1	120.5 (2)
C5—C4—H4	118.9	C16—C15—Cl1	117.8 (2)
C4—C5—C6	121.1 (2)	C17—C16—C15	118.8 (3)
C4—C5—C10	119.1 (2)	C17—C16—H16	120.6
C6—C5—C10	119.7 (2)	C15—C16—H16	120.6
C7—C6—C5	122.0 (2)	C16—C17—C12	120.8 (2)
C7—C6—H6	119.0	C16—C17—H17	119.6
C5—C6—H6	119.0	C12—C17—H17	119.6
C6—C7—C8	119.5 (2)	O2—C18—H18A	109.5
C6—C7—H7	120.3	O2—C18—H18B	109.5
C8—C7—H7	120.3	H18A—C18—H18B	109.5
O3—C8—C9	116.5 (2)	O2—C18—H18C	109.5
O3—C8—C7	123.9 (2)	H18A—C18—H18C	109.5
C9—C8—C7	119.5 (2)	H18B—C18—H18C	109.5
C8—C9—C10	122.6 (2)	O3—C19—H19A	109.5
C8—C9—Br1	116.03 (17)	O3—C19—H19B	109.5
C10—C9—Br1	121.22 (17)	H19A—C19—H19B	109.5
C9—C10—C5	116.3 (2)	O3—C19—H19C	109.5
C9—C10—C1	125.5 (2)	H19A—C19—H19C	109.5
C5—C10—C1	118.2 (2)	H19B—C19—H19C	109.5
O1—C11—C12	120.8 (2)		
			
C18—O2—C2—C1	162.1 (2)	C4—C5—C10—C9	177.4 (2)
C18—O2—C2—C3	−16.0 (4)	C6—C5—C10—C9	−4.2 (3)
C10—C1—C2—O2	−179.2 (2)	C4—C5—C10—C1	−4.0 (3)
C11—C1—C2—O2	−14.2 (3)	C6—C5—C10—C1	174.3 (2)
C10—C1—C2—C3	−1.2 (3)	C2—C1—C10—C9	−177.6 (2)
C11—C1—C2—C3	163.9 (2)	C11—C1—C10—C9	18.6 (3)
O2—C2—C3—C4	176.3 (3)	C2—C1—C10—C5	4.0 (3)
C1—C2—C3—C4	−1.6 (4)	C11—C1—C10—C5	−159.8 (2)
C2—C3—C4—C5	1.6 (4)	C2—C1—C11—O1	−92.9 (3)
C3—C4—C5—C6	−177.0 (3)	C10—C1—C11—O1	71.6 (3)
C3—C4—C5—C10	1.3 (4)	C2—C1—C11—C12	82.9 (3)
C4—C5—C6—C7	179.2 (3)	C10—C1—C11—C12	−112.7 (2)
C10—C5—C6—C7	0.9 (4)	O1—C11—C12—C17	−3.6 (3)
C5—C6—C7—C8	2.5 (4)	C1—C11—C12—C17	−179.3 (2)
C19—O3—C8—C9	−179.9 (2)	O1—C11—C12—C13	177.3 (2)
C19—O3—C8—C7	1.1 (3)	C1—C11—C12—C13	1.6 (3)
C6—C7—C8—O3	176.7 (2)	C17—C12—C13—C14	0.1 (4)
C6—C7—C8—C9	−2.3 (3)	C11—C12—C13—C14	179.2 (2)
O3—C8—C9—C10	179.58 (19)	C12—C13—C14—C15	−0.7 (4)
C7—C8—C9—C10	−1.3 (3)	C13—C14—C15—C16	0.9 (4)
O3—C8—C9—Br1	−4.0 (3)	C13—C14—C15—Cl1	179.9 (2)
C7—C8—C9—Br1	175.04 (16)	C14—C15—C16—C17	−0.4 (4)
C8—C9—C10—C5	4.5 (3)	Cl1—C15—C16—C17	−179.43 (19)
Br1—C9—C10—C5	−171.66 (16)	C15—C16—C17—C12	−0.2 (4)
C8—C9—C10—C1	−173.9 (2)	C13—C12—C17—C16	0.4 (4)
Br1—C9—C10—C1	9.9 (3)	C11—C12—C17—C16	−178.7 (2)

### Hydrogen-bond geometry (Å, °)

**Table:**

Cg1 is the centroid of the C1–C5/C10 ring.

**Table:**

*D*—H···*A*	*D*—H	H···*A*	*D*···*A*	*D*—H···*A*
C4—H4···O1^i^	0.95	2.49	3.366 (3)	154
C19—H19A···Br1^ii^	0.98	2.92	3.871 (3)	165
C19—H19C···O1^iii^	0.98	2.53	3.211 (3)	126
C19—H19B···Cg1^iv^	0.98	2.70	3.509 (3)	140

Symmetry codes: (i) *x*, −*y*+3/2, *z*+1/2; (ii) −*x*, −*y*, −*z*+1; (iii) *x*, −*y*+1/2, *z*+1/2; (iv) −*x*, −*y*+1, −*z*+1.
